# Risk factors related balance disorder for patients with dizziness/vertigo

**DOI:** 10.1186/s12883-021-02188-7

**Published:** 2021-05-08

**Authors:** Zhentang Cao, Cuiting Zhu, Yanan Zhou, Yan Wang, Meimei Chen, Yi Ju, Xingquan Zhao

**Affiliations:** 1 Department of Neurology, Beijing Tiantan Hospital, Capital Medical University, No.119, South 4th Ring West Road, Fengtai District, Beijing, 100070 China; 2 China National Clinical Research Center for Neurological Diseases, Beijing Tiantan Hospital, Capital Medical University, Beijing, China; 3 Clinical Center for Vertigo and Balance Disturbance, Beijing Tiantan Hospital, Capital Medical University, Beijing, China; 4 Research Unit of Artificial Intelligence in Cerebrovascular Disease, Chinese Academy of Medical Sciences, Beijing, China

**Keywords:** Atherosclerosis, Dizziness, Vertigo, Balance, Fall, Nystagmus

## Abstract

**Background:**

When dizziness/vertigo patients presented with balance disorder, it will bring severe morbidity. There is currently lack of research to explore risk factor related balance disorder in dizziness patients, especially in those who walk independently.

**Aim:**

To investigate risk factors related balance disorder in dizziness/vertigo patients who walk independently.

**Methods:**

Medical data of 1002 dizziness/vertigo patients registered in vertigo/balance disorder registration database were reviewed. The demographic data, medical history, and risk factors for atherosclerosis (AS) were collected. Enrolled dizziness/vertigo patients could walk independently, completed Romberg test, videonystagmography (VNG), and limits of stability (LOS). The subjective imbalance was patient complained of postural symptom when performing Romberg test. Multivariable logistic regression analyzed risk factors related balance disorder. The receiver operating characteristic (ROC) curve evaluated the utility of regression model.

**Results:**

Five hundred fifty-three dizziness/vertigo patients who walk independently were included in the final analysis. According to LOS, patients were divided into 334 (60%) normal balance and 219 (40%) balance disorder. Compared with normal balance, patients with balance disorder were older (*P* = 0.045) and had more risk factors for AS (*P*<0.0001). The regression showed that risk factors for AS (OR 1.494, 95% CI 1.198–1.863), subjective imbalance (OR 4.835, 95% CI 3.047–7.673), and abnormality of optokinetic nystagmus (OR 8.308, 95% CI 1.576–43.789) were related to balance disorder. The sensitivity and specificity of model were 71 and 63% (*P*<0.0001). The area under the curve (AUC) was 0.721.

**Conclusions:**

Risk factors for AS, subjective imbalance, and abnormality of optokinetic nystagmus were predictors for balance disorder in patients with dizziness/vertigo who walk independently.

## Background

The vestibular system plays a significant role in perceiving body motion, maintaining balance, postural control, and ocular motor control [1]. Dizziness and imbalance are the typical symptoms of vestibular dysfunction. Dizziness/vertigo is one of the most frequent chief complaints in the neurological clinic, accounting for 5% in the outpatient [2] and 4% in the emergency department consultation [3]. The population-based studies indicated that the prevalence of dizziness was from 17 to 30% [4]. Dizziness/vertigo and imbalance are also the most predominant symptoms in the elderly. The annual prevalence for serious dizziness that affects daily life is 20% in older than 60, 30% in those over 70, and 50% older than 80 [5].

The maintenance of balance function requires integrating of multiple systems, including visual, somatosensory, and vestibular systems. The causes of balance disorders are complicated. The spectrum of etiology covers various diseases. When the dizziness patients present with balance disorder, it will bring many severe morbidities such as fall [6, 7]. Currently, fall has already become a troublesome and threatening public health issue. It can lead to many serious consequences such as fracture, mobility restriction, decreased daily life ability, and mood disorder. Even fall can cause life-threating injuries, especially in the elderly [8]. These related injuries often lead to hospitalization and nursing home admission, and bring large medical expenditures [9]. Meanwhile, dizziness and balance disorder also reduce life quality and bring huge economic burden [10].

The previous studies pay more attention to the specific disease. Some dizziness patients who can walk independently without obvious imbalance symptoms are often neglected. There is currently a lack of research to explore the risk factors related to these dizziness patients. Meanwhile, based on the above various serious complications resulting from dizziness and balance disorder, it is significant to identify risk factors related balance disorder.

Therefore, we aimed to investigate the risk factors related balance disorder in dizziness patients who can walk independently in order to early recognition and treatment.

## Methods

### Study design and participants

**Fig. 1:**
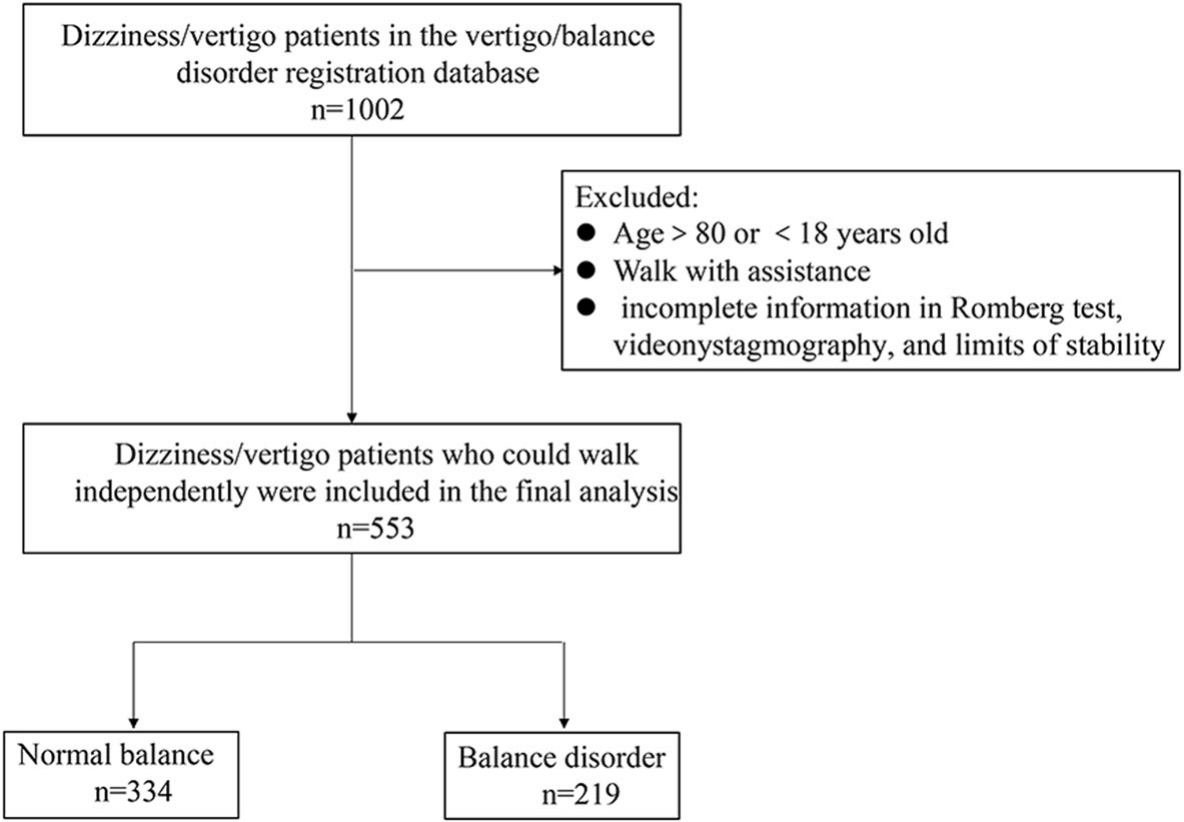
Flowchart of this study

The study was approved by Ethics Committee of Beijing Tainan Hospital and performed according to the Declaration of Helsinki guidelines. Informed consent was obtained from the participants. We reviewed the medical data of 1002 dizziness/vertigo patients who registered in vertigo/balance disorder registration database of the Clinical Center for Vertigo and Balance Disturbance of Beijing Tiantan Hospital. The definition of dizziness and vertigo was in accordance with the Bárány Society consensus [11]. Dizziness is the sensation of disturbed or impaired spatial orientation without a false or distorted sense of motion. Vertigo is the sensation of self-motion (of head/body) when no self-motion is occurring or the sensation of distorted self-motion during an otherwise normal head movement. The eligible patients who met the following criteria for inclusion in this study were: 1) 18 ≤ age ≤ 80 years; 2) complaint of dizziness or vertigo symptom; 3) ability to walk independently without assistance; 4) completed Romberg test, videonystagmography (VNG) and limits of stability (LOS) of computed dynamic posturography (CDP). Exclusion criteria were as follows: 1) contraindication of VNG; 2) any disease history that precludes patients from evaluating of balance state and vestibular function including dementia, visual impairment, acute stroke, movement disorders, musculoskeletal disease, and disease of extraocular muscle. The detailed flowchart figure was shown in the Fig. 1.

### Demographic and clinical characteristics

Comprehensive history taking questionnaires including demographic and clinical characteristics were performed on all participants. The demographic information was consisted of age and gender. The clinical characteristics referred to symptoms, medical history, and risk factors for atherosclerosis (AS). The medical history included benign paroxysmal positional vertigo (BPPV), Ménière’s disease (MD), sudden deafness, vestibular neuronitis (VN), motion sickness, migraine, hyperthyroidism, anemia, orthostatic hypotension, obstructive sleep apnea-hypopnea syndrome (OSAHS), and traumatic brain injury (TBI). The AS risk factors included age ≥ 60 years, hypertension, diabetes, hyperlipidemia, coronary heart disease (CHD), and stroke.

### Neurological examination and Romberg test

All participants underwent detailed neurological examination including consciousness, speech, cranial nerve, sensory, and motor. Cerebellum function was assessed: index-nose test, and heel-knee test. Meanwhile, we also performed Romberg test which can reflect the functions of somatosensory system, cerebellum function, and muscular strength. The participants were asked to maintain balance in the natural standing position with feet together and arms beside the body. The eyes were closed. The definition of subjective imbalance was patient's complained of postural symptoms (one of unsteadiness, directional pulsion, balance-related near fall, and balance-related fall) when performing the Romberg test.

### Vestibular function evaluation-VNG

All enrolled patients adopted VNG to evaluate the vestibular function. The VNG test (Micro-medical, VisualEyes 20,178,624, America) included spontaneous nystagmus test, gaze test, saccade test, smooth pursuit test, head shaking test (HST), optokinetic nystagmus test, positioning test (Roll test or Dix-Hallpike test), and caloric test. To test spontaneous nystagmus, the subject was seated in the upright position with looking straight ahead. The eye movements were recorded for at least 20–30 s under two conditions: open eyes in normal bright room and dark room. The subject looked at the four eccentric optotypes in turn, respectively 30 degrees on the left/right and up/down 25 degrees to test gaze. Each direction was recorded for at least 20 s. When nystagmus occurred, the observation record was 60s. Spontaneous nystagmus with slow phase velocity (SPV) >3°/sec and gaze-evoked nystagmus >6°/sec were considered abnormal. To test saccade, the subject took a sitting position with head fixed in the middle position, and eyes were 1.2 m away from the visual target. The subject was asked to look at the target and record their eye movements. Each test lasts at least 1 min. The subject looked at and swing in a sine wave with the visual target. The smooth pursuit test was divided into four types according to the eye movement curve: I and II were considered normal, III and IV were abnormal. To test optokinetic nystagmus, firstly the subject was instructed to gaze at a fixed spot projected on the center of a uniform white hemispherical full- field screen (diameter 140 cm) in a dark room. A random dot pattern (black and white with uniform acceleration and deceleration) was projected (ranging in size from 0.5 to 2.0 cm in diameters, covering 87% of the visual field) on the screen. The subject was presented with a pattern that filled the greater part of his peripheral and foveal ranges of vision. The subjects were in sitting position, head fixed, facing the horizontal target in front of them. The subjects were asked to keep an eye on the target and count the number of targets silently. The subjects were exposed optokinetic stimulation at speeds of 60 degrees/s preceded by acceleration at 6 degrees/s^2^. Bilateral asymmetry was considered abnormal. HST was performed with the patient sitting in a clinical chair with the head leaning down by 30°. The patient’s head was vigorously rotated for 20 times on the horizontal plane with a maximum amplitude around 30–40°. Post HST nystagmus was recorded for 1 min and was considered positive when nystagmus lasting at least 5 s was detected. The HST was performed to evaluate the vestibulo-ocular reflex, which represents the control of eye movements by the vestibule. A peripheral etiology was related to horizontal or horizontal-rotatory nystagmus, indicative of a vestibulo-ocular pathway abnormality. The supine-roll test was performed with the neck flexed forward about 30° in order to bring the lateral semicircular canal into alignment with the vertical. Then examiner turned the patient’s head to the right or left in the supine position, and eye movements were recorded for 30–60 s. To test Dix-Hallpike, patient sited upright. The examiner turned the patient’s head 45 degrees to the patient’s right. Then examiner quickly changed the posture from sitting position to the supine position with the head hanging below the top end of the examination table at an angle of 30 degrees. The test was abnormal if three or more than beats of nystagmus were observed. BPPV patients showed a few seconds of the incubation period, short-term dizziness and vertical rotation nystagmus occurrence. Caloric test was performed with the head supine and tilted forward 30°. Two methods were applied to perform caloric test. One irrigated the right and left ears with cold water (30 °C) and hot water (44 °C). The other irrigated the right and left ears, respectively with cold air (24 °C) and hot air (50 °C). The canal paresis (CP) value greater than 25% was defined as abnormal according to the Jongkees Index formula calculation.

### Dynamic balance function evaluation-LOS

The dynamic balance function evaluation was performed using the SMART EquiTest balance assessment system (NeuroCom International, Clackamas, OR, USA). The LOS test can evaluate an individual’s ability to move their center of gravity (COG) to the given eight direction in space. There is a stick figure in the system screen representing the subject’s live-time dynamic center of pressure (COP). The figure is surrounded by eight targets that represent the maximal theoretical stability of patients. When the COP of patients moves, the figure on the screen will move accordingly. Once the voice prompt sounds, subjects need to move their body to the target that appears on the screen and keep balance as much as possible. A total of eight trials were measured, each with 8 s. Every trial was incorporated in the equation of the following measurements, which are mean composite (eight directions/trials averaged) scores: 1) Reaction time (RT) is the time from the start of trial to patient’s first movement (seconds); 2) Movement velocity (MVL) is the mean speed of COG movement (degrees/second); 3) Endpoint excursion (EXE) is the distance of COP displacement when initial lean towards target (% of maximal LOS); 4) Maximum excursion (MXE) is farthest distance of COP displacement during the eight trials (% of maximal LOS); 5) Directional control (DCL) is a comparison of the amount of movement in the planned direction (towards the target) to the amount of extraneous movement (away from the target). When the MVL, DCL, one of the EXE and MXE were both abnormal according to the results of LOS, we considered it as balance disorder.

### Statistical analysis

Continuous variable namely age was described as mean ± standard deviation (SD). Categorical variables were presented as frequency and percentage. Baseline characteristics between normal balance and balance disorder were compared with chi-squared test for categorical and Mann-Whitney U test for age. Multivariable logistic regression analysis was adopted to analyze the risk factors related balance disorder. We incorporated the variable of *P*<0.2 in the univariate analysis into the multivariable analysis. The receiver operating characteristic (ROC) curve was performed to evaluate sensitivity and specificity of regression model. The “optimum” cut-off value for ROC curve was defined as the value associated with the maximal sum of sensitivity and specificity. Statistical analysis was performed in SPSS 24.0 (IBM, Chicago, IL, USA). Values with *P* < 0.05 were regarded as statistically significant.

## Results

### Baseline characteristics of participants

We reviewed 1002 dizziness/vertigo patients who registered in the vertigo/balance disorder registration database of Clinical Center for Vertigo and Balance Disturbance of Beijing Tiantan Hospital from Jul 2019 to Jul 2020. After excluding patients with incomplete clinical data, a total of 553 patients who could walk independently were included in the final analysis. Five hundred fifty-three patients both completed VNG and LOS examinations. According to abnormal results of LOS, participants were divided into 334 (60%) normal balance and 219 (40%) balance disorder.

**Fig. 2:**
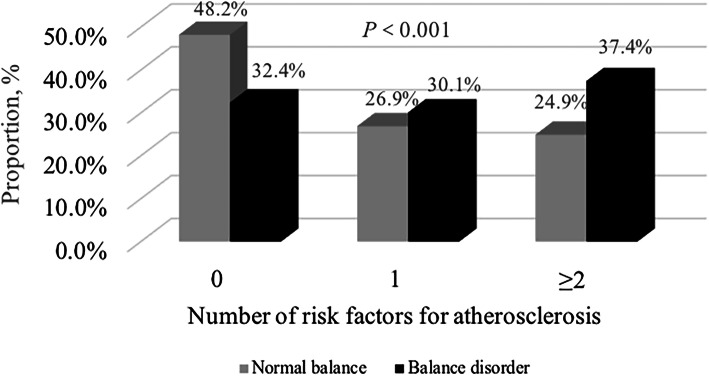
The distribution of number of risk factors for atherosclerosis between normal balance and balance disorder groups

The demographics and baseline clinical characteristics were shown in Table 1. There was no significant difference in sex between normal balance and balance disorder groups. Compared to patients with normal balance group, patients with balance disorder were older (*P* = 0.045) and had more risk factors for atherosclerosis (*P*<0.0001, Fig. 2). When we performed the Romberg test, more patients in the balance disorder group complaint of imbalance by verbal report to the physician (*P*<0.0001). No significant difference was found in other medical history which was related to dizziness/vertigo symptom between two groups.

**Table 1:** Demographic and baseline characteristics in patients with dizziness/vertigo according to balance status

Variables	Total (*N* = 553)	Normal balance (*N* = 334)	Balance disorder (*N* = 219)	*P* value
Age, y	53.6 ± 12.59	52.98 ± 11.83	54.56 ± 13.65	0.045
Male, n (%)	216(39.1)	137(41.0)	79(36.1)	0.244
Subjective imbalance, n (%)	381(68.9)	192(57.5)	189(86.3)	<0.0001
Medical history, n (%)				
BPPV	7(1.3)	5(1.5)	2(0.9)	0.540
MD	10(1.8)	4(1.2)	6(2.7)	0.190
Sudden deafness	8(1.4)	4(1.2)	4(1.8)	0.549
VN	1(0.2)	1(0.3)	0(0.0)	0.315
Motion sickness	139(25.1)	77(23.1)	62(28.3)	0.163
Migraine	23(4.2)	15(4.5)	8(3.7)	0.629
Hyperthyroidism	3(0.5)	1(0.3)	2(0.9)	0.343
Anemia	11(2.0)	7(2.1)	4(1.8)	0.824
Orthostatic hypotension	2(0.4)	1(0.3)	1(0.5)	0.766
OSAHS	4(0.7)	3(0.9)	1(0.5)	0.537
Traumatic brain injury	13(2.4)	8(2.4)	5(2.3)	0.932
Number of risk factors for AS				<0.0001
0	232(42.0)	161(48.2)	71(32.4)	
1	156(28.2)	90(26.9)	66(30.1)	
≥ 2	165(29.8)	83(24.9)	82(37.4)	

*Abbreviations*: *LOS* limits of stability, *BPPV* benign paroxysmal positional vertigo, *MD* Ménière’s disease, *VN* Vestibular neuronitis, *OSAHS* obstructive sleep apnea-hypopnea syndrome, *AS* atherosclerosis

### Comparison of VNG results according to the balance status

All included patients had received the VNG examination. The detailed results were indicated in Table 2. The abnormality of optokinetic nystagmus test was more common in the balance disorder group (4.1% vs 0.6%, *P* = 0.004). Other testes of VNG including saccade, spontaneous nystagmus, gaze, smooth pursuit test, head shaking, positioning, and caloric testes, didn’t have significant difference between two groups.

**Table 2:** Comparison of abnormal VNG in patients according to the balance status

Variables	Total (*N* = 553)	Normal balance (*N* = 334)	Balance disorder (*N* = 219)	*P* value
Saccade test, n (%)	6(1.1)	3(0.9)	3(1.4)	0.605
Spontaneous nystagmus, n (%)	20(3.6)	10(3.0)	10(4.6)	0.333
Gaze test, n (%)	13(2.4)	7(2.1)	6(2.7)	0.625
Smooth pursuit test, n (%)	10(1.8)	4(1.2)	6(2.7)	0.190
Optokinetic test, n (%)	11(2.0)	2(0.6)	9(4.1)	0.004
Head shaking test, n (%)	79(14.3)	49(14.7)	30(13.7)	0.749
Positioning test, n (%) ^*^	68(12.3)	35(10.5)	33(15.1)	0.108
Caloric test, n (%) ^*^	122(22.1)	73(21.9)	49(22.4)	0.886

*Abbreviations*: *VNG* videonystagmography

^*^Positioning test referred to supine-roll test or the Dix-Hallpike

^*^In the abnormal caloric test, there was 86 patients who performed air caloric test and 36 patients performed water caloric test

### Risk factors analysis for balance disorder

**Fig. 3:**
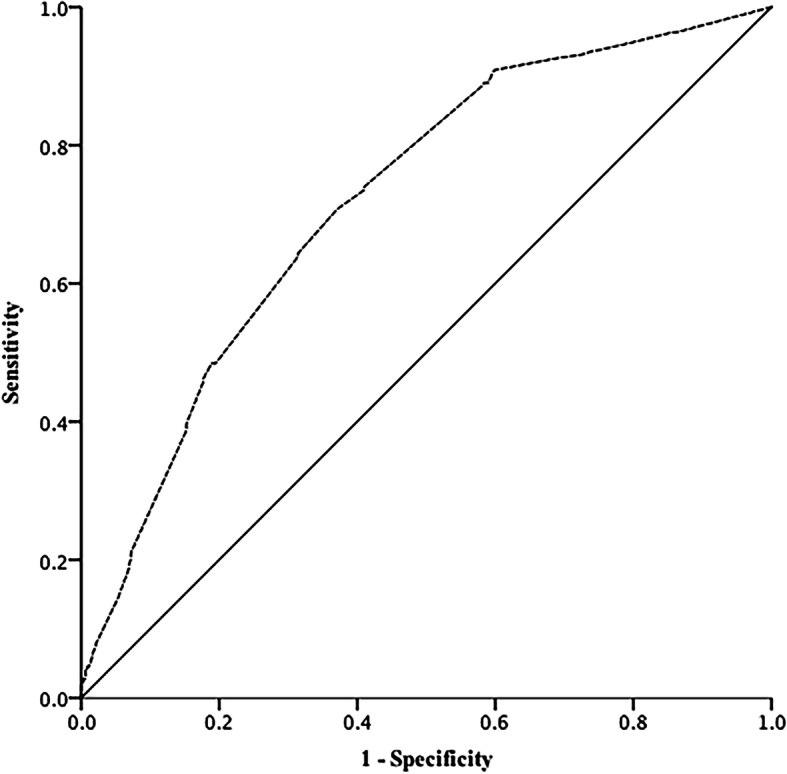
ROC curve for the diagnosis of balance disorder for dizziness/vertigo patients who walk independently

Based on the results of univariable analysis, we performed multivariable analyses to investigate the risk factors for balance disorder in patients with dizziness/vertigo who could walk independently. The consequences were shown in Table 3. The results showed that subjective imbalance (OR 4.835, 95% CI 3.047–7.673, *P*<0.0001), abnormality of optokinetic nystagmus (OR 8.308, 95% CI 1.576–43.789, *P* = 0.013), and risk factors for AS (OR 1.494, 95% CI 1.198–1.863, *P*<0.0001) were associated with balance disorder. The smooth pursuit test, positioning test, medical history of MD and motion sickness were not related to balance disorder. To evaluate the utility of regression model in identifying the risk factors for balance disorder, ROC curve was depicted in Fig.3. The sensitivity and specificity of regression model were 71 and 63% respectively (95% CI 0.678–0.764, *P*<0.0001). The area under the curve (AUC) was 0.721.

**Table 3:** Risk factors associated with balance disorder in the dizziness patients

Variables	OR	95% CI	*P* value
Subjective imbalance	4.835	3.047–7.673	<0.0001
Optokinetic nystagmus	8.308	1.576–43.789	0.013
Smooth pursuit test	2.190	0.531–9.036	0.278
Positioning test^*^	1.324	0.771–2.273	0.309
Risk factors for AS	1.494	1.198–1.863	<0.0001
MD	3.607	0.897–14.508	0.071
Motion sickness	1.343	0.880–2.050	0.171

*Abbreviations*: *AS* atherosclerosis, *MD* Ménière’s disease

^*^Positioning test referred to supine-roll test or the Dix-Hallpike

## Discussion

In this study, patients with balance disorders had more risk factors for atherosclerosis. And risk factors for AS, subjective imbalance, and abnormality of optokinetic nystagmus were predictors for balance disorder in patients with dizziness/vertigo who can walk independently without assistance.

The novelty in this study was study population. We focused on the dizziness patients who could walk independently without assistance. Clinically, this part of the population is often ignored. The potential risk for balance disorder of this group is unknown. In our study, the prevalence of balance disorder in patients with dizziness/vertigo who can walk independently without assistance is 40%. This prevalence was very high. Balance disorder is closely associated with some adverse outcomes such as fall and even death [7]. Therefore, it is significant to identify vulnerable people and explore predictors for balance disorder before the occurrence of detrimental events.

The risk factors for AS were predictors for balance disorder in patients with dizziness/vertigo who can walk independently without assistance in our study. The AS risk factors included age ≥ 60 years, hypertension, diabetes, hyperlipidemia, coronary heart disease (CHD), and stroke. These factors can both affect the balance function. Our results showed dizziness patients with the balance disorder were older than that without balance disorder. The maintenance of balance function requires the integration of multiple organ systems such as vestibular, proprioceptive, visual, musculoskeletal, cardiovascular systems and so on. The above function of each system can degenerate with aging [12]. Especially in the vestibular function, age-related changes include the degeneration of otoconia, loss of vestibular afferents and hair cells in the labyrinth [13, 14]. At the same time, most remarkable effect of aging on the musculoskeletal system was muscle strength [15] which can lower by 20–40% in the over 70 years old compared with young adults. The vibration and touch thresholds are also decreased. The tactile information between ground and feet is insensitive. And the perception ability of position and direction declined with aging [16]. These age-related changes will affect the proprioceptive system. As for visual system, aging is associated with visual acuity and dark adaption [17]. Therefore, the patients with balance disorder may be older. Certainly, it needs the further study to investigate the balance function according to the age status.

Other risk factors for AS also can affect the balance function. The chronic hypertension could damage the large arteries and micro-circulation in specific functional areas related with balance. At the same time, it is the leading risk factor for white matter lesions (WML), which can be found in the periventricular region and in the brainstem, which can damage the inter-neural connections [18]. Further, WML, periventricular lesions, and brain stem lesions were associated with impaired balance [19]. The diabetes often has various complications such as diabetic retinopathy or peripheral neuropathy. These complications can damage visual, and somatosensory systems of the body [20]. The impairment of any one of these systems can cause a feeling of instability. The most widely impairment caused by stroke is motor damage which may severely impact gait and balance [21]. Therefore, the more risk factors for atherosclerosis, the more likely has balance disorders. This is consistent with our results.

Our study also showed the subjective imbalance was a predictor for balance disorder in patients with dizziness. The definition of subjective imbalance is patients who complained of postural symptoms when performing Romberg test. The Romberg test can show the patients’ ability to keep balance. It can reflect the integral function of cerebellum and proprioception. Meanwhile, it also can indirectly reflect the muscular strength. This included elements of a wide range of brain functions, such as cognition and emotion. Therefore, patients who complain of subjective imbalance are more likely to have balance disorder. Meanwhile, history taking is an important part for the diagnosis of vestibular disorder [22]. Clinically, when we perform some examinations, we are prone to neglect the patients’ feeling. The communication between patients and doctors is lacking. But sometimes some complaints may be the red flags for disease progression. From our point of view, we should pay more attention to patients’ sensation especially during the examination.

The optokinetic nystagmus (OKN) is a reflexive eye movement that makes the eyes to follow an object within a visual field with a slow tracking movement (slow phase) followed by a rapid resetting movement (saccade) (quick phase) [23]. The “look” and “stare” OKN were two forms of OKN. OKN is significant to keep a stable retinal image during the head movement relative to the environment. Our study showed that abnormal OKN was associated with balance disorder. This phenomenon might be explained by the complicated pathway of OKN. The pathway included cortical, subcortical, and infratentorial ocular motor pathways. The main brain regions include the cortical eye fields (frontal and supplementary), the prefrontal, and the visual cortex [24, 25]. Meanwhile, in addition to the higher level cortical ocular motor areas, several functional imaging studies indicated that brainstem and cerebellum also take part in the occurrence of OKN [25–27]. As we known, these brain regions also take part in the postural and gait control [28]. The disorder of these brain regions can cause balance disorder. This also shows that OKN’s abnormality can indirectly reflect balance disorders.

This was the first study to investigate risk factors related balance disorder in dizziness patients who can walk independently. It was helpful to early recognition and treatment for balance disorder. But our study still had some limitations. Firstly, we didn’t perform the detailed evaluations involving visual system, somatosensory system, cognition, and emotion, such as vision and somatosensory evoked potential. But this is the initial study to investigate risk factors related balance disorder in patients with dizziness. In the future, we will combine the present results with more detailed evaluations. Secondly, we didn’t analyze the imaging characteristics of dizziness patients. This is because relatively few patients completed the imaging examination. Further we will take the features of imaging into our analysis.

## Conclusions

Risk factors for AS, subjective imbalance, and abnormality of optokinetic nystagmus were predictors for balance disorder in patients with dizziness/vertigo who can walk independently.

## Data Availability

The datasets generated for this study are available on request to the corresponding author.

## Abbreviations

VN: Vestibular Neuronitis
VNG: Videonystagmography
LOS: Limits of stability
CDP: Computed dynamic posturography
AS: Atherosclerosis
BPPV: Benign paroxysmal positional vertigo
MD: Ménière’s disease
OSAHS: Obstructive sleep apnea-hypopnea syndrome
TBI: Traumatic brain injury
CHD: Coronary heart disease
SPV: Slow phase velocity
CP: Canal paresis
COG: Center of gravity
COP: Center of pressure
RT: Reaction time
MVL: Movement velocity
EXE: Endpoint excursion
MXE: Maximum excursion
DCL: Directional control
ROC: Receiver operating characteristic
OKN: Optokinetic nystagmus
